# Dietary Neutral Detergent Fiber Levels Impacting Dairy Cows’ Feeding Behavior, Rumen Fermentation, and Production Performance during the Period of Peak-Lactation

**DOI:** 10.3390/ani13182876

**Published:** 2023-09-10

**Authors:** Renhuang Shi, Shuangzhao Dong, Jiang Mao, Jingjun Wang, Zhijun Cao, Yajing Wang, Shengli Li, Guoqi Zhao

**Affiliations:** 1 Beijing Engineering Technology Research Center of Raw Milk Quality and Safety Control, State Key Laboratory of Animal Nutrition, Department of Animal Nutrition and Feed Science, College of Animal Science and Technology, China Agricultural University, Beijing 100094, China; srh021339@126.com (R.S.); 18813060688@163.com (S.D.); maojiang@newhope.cn (J.M.); wangjingjun@cau.edu.cn (J.W.); caozhijun@cau.edu.cn (Z.C.); yajingwang@cau.edu.cn (Y.W.); 2 College of Animal Science and Technology, Yangzhou University, Yangzhou 225009, China; gqzhao@yzu.edu.cn

**Keywords:** neutral detergent fiber, dairy cows, rumen fermentation, production performance

## Abstract

**Simple Summary:**

Dietary neutral detergent fiber (NDF) is commonly used as an indicator of the fiber content in animal diets, particularly in ruminant nutrition. NDF plays a crucial role in the digestive process of ruminant animals, as it affects feed intake, rumen fermentation, and nutrient utilization. The levels of NDF affect feeding behavior, rumen fermentation, and production performance during the period of peak lactation in dairy cows. To prove it, four Holstein dairy cows were subjected to a feeding experiment with varying NDF levels in their diets. The results indicated that increased NDF levels resulted in reduced dry matter intake, while the time spent eating and ruminating increased. Moreover, higher NDF levels led to increased pH value and acetate concentration, as well as changes in the proportions of certain bacteria in the rumen. Milk yield, protein percentage, and nitrogen efficiency decreased with increasing NDF levels, while milk fat percentage and milk urea nitrogen concentration increased. Notably, diets with 25% and 34% NDF had negative effects on feeding behavior, rumen fermentation, and production performance. However, the diet with 28% NDF was effective in enhancing the production performance compared to the one with 31% NDF. These findings offer a valuable strategy for optimizing the provision of dietary NDF to cattle, thereby enhancing their overall performance.

**Abstract:**

This study investigated the impact of dietary neutral detergent fiber (NDF) levels (25.49%, 28.65%, 31.66%, and 34.65%, respectively) on the feeding behavior, rumen fermentation, cellulolytic bacteria, and production performance of dairy cows during peak lactation. A feeding experiment was conducted using four fistulated Holstein dairy cows (600 ± 25 kg) with days in milk (50 ± 15 days), employing a 4 × 4 Latin square design to assign the cows to four groups. The results demonstrated that increasing NDF levels in the diet had the following effects: (1) A linear decrease in dry matter intake (DMI), NDF intake, and physically effective NDF_8.0_ (peNDF_8.0_) intake; a linear increase in the average time spent eating and ruminating, as well as the time spent eating and ruminating per kilogram of dry matter (DM); a quadratic response in the time spent ruminating per kilogram of NDF and peNDF_8.0_. (2) A linear increase in average pH value, acetate concentration, and the proportions of Fibrobacter succinogenes and Ruminococcus flavefaciens among total bacteria; a linear decrease in ammonia nitrogen (NH_3_-N) concentration, microbial crude protein (MCP), total volatile fatty acid (TVFA), propionate, butyrate, and lactate. (3) A linear decrease in milk yield, milk protein percentage, and nitrogen efficiency of dairy cows; a linear increase in milk fat percentage and milk urea nitrogen (MUN) concentration. Based on the combined results, it was found that diets with 25% and 34% NDF had detrimental effects on the feeding behavior, rumen fermentation, and production performance of dairy cows. However, the diet with 28% NDF showed superior outcomes in production performance compared to the one with 31% NDF. Therefore, it is strongly recommended to include a diet containing 28% NDF during the critical peak lactation period for dairy cows.

## 1. Introduction

In China, the selection of roughage for inclusion in dairy cow diets predominantly consists of alfalfa hay, oat hay, Leymus chinensis, corn silage, and rice straw [1]. These roughage options are widely utilized due to their nutritional value and availability within the region. Effective and rational utilization of high-quality forage in dairy farm management not only ensures the health of dairy cows but also improves their production performance [2,3]. The diet serves as the primary energy source for maintaining normal physiological functions in dairy cows, and the presence of dietary neutral detergent fiber (NDF), particularly in roughage, is a crucial nutritional indicator for ruminants. Numerous studies have demonstrated its significance in stimulating saliva secretion, promoting rumination, maintaining rumen health, and ensuring optimal production performance in dairy cows [4,5]. Moreover, an increase in NDF content in the diet has been associated with a linear reduction in dry matter intake (DMI) [6,7,8].

The National Research Council (NRC 2001) recommends an NDF level of approximately 25–30% of dry matter in the rations of lactating dairy cows, with roughage-derived NDF accounting for 19% [9]. Currently, there is a growing pressure to meet the production performance, health, and welfare requirements of dairy cows. However, there is no specific optimal concentration of NDF recommended under different lactation levels for dairy farms, especially for Chinese dairy group companies. The peak lactation period is of particular importance; the report showed that every added kilogram (kg) of peak milk could lead to 200 to 250 kg more milk for the whole lactation period of the cow [10]. However, there is no precise recommended value for the appropriate NDF level during this physiological stage [11]. Therefore, it is imperative to establish and enhance the research on NDF requirement parameters in the dry matter of dairy cow diets according to Chinese feeding standards.

The primary aim of this study is to comprehensively evaluate the impact of different levels of dietary NDF on various aspects of Chinese Holstein dairy cows during the peak lactation period. This study encompasses a wide range of parameters, including feeding behavior, rumen fermentation, cellulolytic bacteria, and production performance, with the objective of determining the optimal NDF requirement specifically for Chinese Holstein dairy cows during this critical stage. By conducting experimental trials and gathering insightful data, this research aims to establish precise guidelines for NDF levels in the diets of these cows. The ultimate goal is to provide practical recommendations that can optimize the cows’ nutritional intake and effectively support their physiological demands throughout the crucial period of milk production. By establishing specific NDF recommendations tailored to the peak lactation period in Chinese Holstein dairy cows, this study seeks to advance the understanding and implementation of effective feeding strategies within the Chinese dairy industry.

## 2. Materials and Methods

### 2.1. Experimental Materials

#### 2.1.1. Experimental Animals

In accordance with the principle of similar parity, lactation days, and milk yield, four lactating, multiparous, cannulated Holstein cows were selected for the experiment. The cows enrolled in the study had an average lactation period of 50 ± 15 days in milk (DIM) and weighed approximately 600 ± 25 kg in body weight (BW) at the beginning of the trial. The average milk yield for the previous week was recorded as 42 ± 3 kg. The experiment and animal procedures were performed according to the Guidelines for Care and Use of Laboratory Animals of China Agricultural University (Beijing, China) and approved by the Animal Ethics Committee of China Agricultural University (Approval No. AW82211202-1-1).

#### 2.1.2. Experimental Feed

In this study, we utilized a variety of NDF feed raw materials to formulate the diets for our experiment. The main sources of NDF feed raw materials, as outlined in Table 1, included corn silage, imported alfalfa hay, local alfalfa hay, Leymus chinensis, and local oat hay. The experiment was designed with a target DMI of 23 kg per day. To achieve this, we divided the experiment into four groups, each with a different level of dietary NDF. The NDF percentages in the diets were 25.49%, 28.65%, 31.66%, and 34.65%. To formulate the diets, we followed the guidelines provided by the Nutrient Requirements of Dairy Cattle Eighth Revised Edition (NASEM 2021) [11]. Considering a body weight of 600 kg, milk yield of 40 kg, milk fat content of 4%, and milk protein content of 3.2% (as indicated in Table 2), we carefully formulated the diets to meet the specific nutrient requirements. Furthermore, the cutting lengths of the imported alfalfa, local alfalfa, and oat hay were maintained at 4 to 6 cm, while the Leymus chinensis was cut to a length of 6 to 8 cm. These cutting lengths were selected to ensure appropriate feeding and intake characteristics.

### 2.2. Experimental Design and Experimental Diet

#### 2.2.1. Feeding and Management

The cows were provided with two feeding sessions per day, occurring at 7:00 and 14:00, along with unrestricted access to water for drinking purposes. They were housed within a free stall loop system and subjected to three milking sessions in a 2 × 16 parallel parlor. These milking sessions took place at specific time intervals: between 7:00 and 7:30, 14:00 and 14:30, and 20:30 and 21:00. To ensure balanced nutrition, a formula was employed to evenly mix four different diets. The feed was dispensed into an automated feeding trough, and the roughage intake control system utilized electronic ear tags to record the DMI of each cow. It is important to note that two open feeding troughs were shared among all cows.

#### 2.2.2. Experimental Design

At the commencement of the experiment, a total of four cows were selected and randomly allocated to one of the four treatment sequences utilizing a 4 × 4 Latin square design. The study was conducted over four distinct periods, with each period spanning 21 days. Prior to the initiation of each period, an 18 d preliminary phase was implemented, followed by a 3 d data collection phase. Throughout the experiment, various parameters, including feed intake, milk yield, and feeding behavior, were diligently recorded on a daily basis. Additionally, samples of rumen fluid, milk, and feed were collected during the final three days of each period.

### 2.3. Sample Collection and Analyses

#### 2.3.1. Recording the Feed Intake and Feeding Behavior 

The automated feeding trough system meticulously recorded the daily consumption of feed and the duration of feeding for each cow (Roughage Intake Control System, RIC, Insentec B.V., Marknesse, The Netherlands). Additionally, the ruminant counter diligently tracked the daily rumination time of the cows (SCR collar, SCR Engineers Ltd., Netanya, Israel). To determine the daily chewing time, the sum of the feeding time and rumination time was calculated.

To assess the efficiency of the cows’ eating habits, the average time spent on eating, ruminating, or chewing per kilogram of DM, NDF, or physically effective neutral detergent fiber (peNDF) intake was computed. peNDF is the product of the physical effectiveness factor (pef) and the NDF content of a feed (i.e., peNDF8.0 = pef8.0 × NDF), where pef_8.0_ is the proportion of particles retained above an 8 mm sieve, which is measured using a Penn State Particle Separator. This involved dividing the total daily time dedicated to each activity (eating and ruminating) by the mean daily intake of DM, NDF, or peNDF over the measurement days, respectively.

#### 2.3.2. Collection, Preservation, and Pretreatment of Rumen Fluid

On the 21st day of each experimental period, samples of rumen fluid (100 mL) were collected from the ventral sac at seven specific time points: prior to the morning meal (0 h) and subsequent to the feedings at 2, 4, 6, 8, 10, and 12 h. Immediately after collection, the pH of each sample was measured (PHS-3E, INESA Scientific Instrument Co., Ltd. Shanghai, China), and the samples were then strained through four layers of cheesecloth. Subsequently, the strained samples were centrifuged at a rotational speed of 4000 r/min for 15 min. The resulting supernatant was collected for the determination of ammonia nitrogen (NH_3_-N), microbial crude protein (MCP), volatile fatty acid (VFA), and lactate concentrations in the rumen fluid. All samples were stored at a temperature of −20 °C until further analysis. The concentration of NH_3_-N was determined using the traditional phenolsodium hypochlorite colorimetry method [12], and the concentration of VFA in the rumen fluid was determined using a gas chromatograph (6890N; Agent Technologies, Avondale, PA, USA), as described by Erwin [13]. For the MCP determination, the method described by Makkar [14] was followed, and the lactic acid concentration was determined using a colorimetric method employing a lactic acid kit (Nanjing Jiancheng Co., Ltd., Nanjing, China).

For the analysis of rumen microflora, the rumen fluid was collected four hours after feeding, and a volume of 5 mL of the resulting supernatant was collected and stored at a temperature of −80 °C. The determination of rumen microflora was performed using a fluorescent quantitative PCR method. The primer design and synthesis are shown in Table 3. The detailed method includes DNA extraction, DNA Agarose Gel electrophoresis, PCR test, and calculation (based on 2^(-delta-delta CT)^ method to calculate the relative expression of rumen bacteria). The percentage of target bacteria is calculated according to the following formula, target bacteria (% total bacteria 16S rDNA) = 2^−(CT target − CT total bacteria)^ × 100, the cycle threshold (CT) target is the CT value of the target bacteria and CT total bacteria is the CT value of the total bacteria.

#### 2.3.3. Collection and Preservation of Feed and Milk

Over the past three days, we have diligently gathered the latest feed and distributed it on a daily basis. To prepare air-dried samples for testing purposes, the diet samples were subjected to a drying process in an oven set at 65 °C for a duration of 48 h. Subsequently, the dried samples were crushed (40 mesh screen) and preserved for further analysis.

To determine the nutrient composition of the experimental diets, we employed wet chemistry methods, as referenced in [15]. The following parameters were assessed using established techniques outlined by the AOAC [16]: dry matter (DM), organic matter (OM), crude protein (CP), crude fat (EE), calcium (Ca), and phosphorus (P). Furthermore, the NDF and acid detergent fiber (ADF) content were determined utilizing the method developed by Van Soest [17]. Lastly, starch analysis was conducted by employing the colorimetric method outlined by Bal [18].

On the 19th day of each experimental period, we proceeded to collect milk samples for analysis. The collection process involved obtaining samples from each milking session, accounting for their proportion relative to the total daily milk yield. Specifically, morning, noon, and evening milk samples were collected in the proportion of 4:3:3, respectively.

To ensure accurate measurements, two plastic pipes were utilized to collect the milk samples. Each pipe collected 50 mL of milk. One of the pipes was preserved with heavy potassium chromate, a chemical used for preservation purposes. The preserved samples were promptly tested for milk composition, including milk fat percentage, milk protein percentage, and lactose percentage (FOSS Milko Scan TM, Beijing Dairy Center, Beijing, China).

Simultaneously, the second pipe containing the milk sample was stored at a temperature of −20 °C. This sample was earmarked for testing milk urea nitrogen (MUN) levels. The MUN analysis was carried out using the method developed by Ekinci and Broderick (Nanjing Jiancheng Co., Ltd., Nanjing, China)., with details to be specified [19].

### 2.4. Data Analysis

The data obtained from the experiment were subjected to analysis using a general linear model (GLM) of a 4 × 4 Latin square design in SAS 9.1 (SAS Institute Inc., Cary, NC, USA). To determine significant differences, multiple Duncan comparisons were conducted. Furthermore, linear and quadratic regression analyses were employed to examine the impact of increasing NDF in the diet. The significance level was set at *p* < 0.05 unless otherwise specified.

The general linear model of statistical variables is:

Yijkl = μ + Pi + Cj+Tk +Sl + STlk + Eijkl.

Yijkl is the dependent variable value of the experimental cow under different diets; μ is the overall mean; Pi is the effect of the experiment period; Cj is the random effect of the experiment cow; Tk is the effect of the diet k treatment; Sl is the Latin Square effect; STlk is the interaction between the Latin square and the dietary treatment; and Eijkl is the random error.

## 3. Results

### 3.1. Effects of Dietary NDF Levels on Feeding Behavior of Dairy Cows during the Period of Peak-Lactation

#### 3.1.1. Effects of Dietary NDF Levels on DM, NDF, and peNDF8.0 Intake in Dairy Cows during Peak Lactation

The results are summarized in Table 4, indicating a significant linear decrease (*p* < 0.05) in the DMI of lactating dairy cows with increasing levels of dietary NDF. Conversely, there was a linear increase (*p* < 0.05) in the intake of NDF and physically effective NDF_8.0_ (peNDF_8.0_). Notably, the DM intake of dairy cows in the 34% NDF group was significantly lower than that of the 25% and 28% groups (*p* < 0.05). Furthermore, significant differences (*p* < 0.05) were observed in the intake of NDF and peNDF_8.0_ among the treatment groups.

#### 3.1.2. Effects of Dietary NDF Levels on Eating and Ruminating Activities of Dairy Cows during Peak Lactation

The findings presented in Table 5 reveal that as the dietary NDF levels increased there was a significant linear increase (*p* < 0.05) in the average time spent eating and ruminating, as well as the combined time spent eating and ruminating per kilogram of DM. No significant difference (*p* > 0.05) was observed in the average time spent eating per kilogram of peNDF_8.0_, although the test group with 31% NDF exhibited a significantly higher value compared to the test group with 34% NDF (*p* < 0.05).

Furthermore, the test groups with 31% and 34% NDF displayed significantly higher average time spent eating per kilogram of NDF than the group with 25% NDF (*p* < 0.05). Interestingly, the average time spent ruminating per kilogram of NDF and peNDF_8.0_ initially decreased and then increased, conforming to a quadratic curve relationship (*p* < 0.05). Notably, the group with 25% NDF exhibited a significantly higher average time spent ruminating per kilogram of peNDF_8.0_ compared to the other three groups.

### 3.2. Impact of Dietary NDF Levels on Rumen Fermentation Parameters in Dairy Cows during Peak Lactation

#### 3.2.1. Dietary NDF Levels Impact on Ruminal pH, NH_3_-N, MCP, VFA, and Lactate in Dairy Cows during Peak Lactation

**Figure 1:**
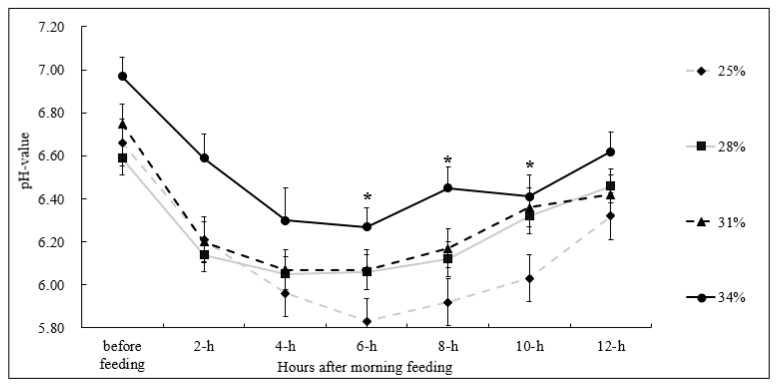
25% NDF group = dashed gray line (♦); 28% NDF group = solid gray line (■); 31% NDF group = dashed black line (▲); 34% NDF group = solid black line (●). Treatment differences (*p* < 0.05) are represented by (*); error bars represent the standard error of the mean.

Based on Figure 1, it can be observed that the rumen fluid pH values of the dairy cows in each experimental group exhibited a consistent pattern following the administration of diets with varying levels of NDF. Initially, there was a decline in pH followed by a subsequent increase, with the lowest pH value being reached approximately 4 to 6 h after feeding. After 8 h, the pH gradually began to rise again. Moreover, it was noted that an increase in the NDF level of the diet led to a linear increase in rumen pH values at six, eight, and ten hours post-feeding (*p* < 0.05). With the increment in dietary NDF level, there was a linear increase in rumen pH values at the six-hour, eight-hour, and ten-hour time points (*p* < 0.05). Moreover, the test group consisting of 25% had significantly lower rumen pH values compared to the test group, comprising 34%.

Table 6 presents the observed trends in response to increasing dietary NDF levels, reflecting various parameters in a professional manner. As the NDF level in the diet increased, the average pH value of the rumen fluid, acetate concentration, and the acetate to propionate ratio displayed a linear increase (*p* < 0.05). Conversely, NH_3_-N, MCP, TVFA, propionate, butyrate, and lactate exhibited a linear decrease (*p* < 0.05). The concentration of caproate showed a linear declining trend, albeit with a *p*-value of 0.06.

Notably, among the test groups, the average pH value of the 34% group was significantly higher compared to the other three groups. Additionally, the 25% and 28% test groups demonstrated significantly lower acetate concentration and acetate to propionate ratio compared to the 31% and 34% test groups. Conversely, the NH_3_-N, MCP, propionate, and lactate levels were significantly higher in the 25% and 28% test groups compared to the 31% and 34% test groups. Furthermore, the TVFA and butyrate concentrations were significantly higher in the 25% test group compared to the 34% test group.

#### 3.2.2. Impact of Dietary NDF Levels on the Proportion of Rumen Cellulolytic Bacteria among Total Bacteria in Dairy Cows during Peak Lactation

Table 7 observes a clear linear increase (*p* < 0.05) in the proportions of Fibrobacter succinogenes and Ruminococcus flavefaciens as the NDF levels in the diet increase. Furthermore, in the test group with a 25% NDF level the proportions of Fibrobacter succinogenes and Ruminococcus flavefaciens were significantly lower compared to the group with a 34% NDF level (*p* < 0.05). Additionally, the proportion of Fibrobacter succinogenes in the 25% test group was significantly lower than that of the 31% test group (*p* < 0.05). However, no significant effects on Ruminococcus albus were observed across the treatments (*p* > 0.05).

### 3.3. Effects of Dietary NDF Levels on Dairy Cows Production Performance during the Period of Peak-Lactation

Table 8 demonstrates the observed effects of increasing dietary NDF levels on various milk production parameters in dairy cows during the peak lactation period. As the NDF levels in the diet increased, a linear decrease (*p* < 0.05) was observed in milk yield, lactose yield, milk protein percentage, milk protein yield, and nitrogen efficiency, and the experiment group with a 34% NDF level exhibited significantly lower values compared to the experiment group with a 25% NDF level (*p* < 0.05). In contrast, there was a linear increase (*p* < 0.05) in milk fat percentage and MUN concentration, with the test group at a 25% NDF level significantly lower than the test group at a 34% NDF level (*p* < 0.05). However, no significant effects (*p* > 0.05) were observed on 4% fat-corrected yield, feed efficiency, and milk lactose percentage.

## 4. Discussion

### 4.1. Effects of Dietary NDF Levels on Dairy Cows Feeding Behavior during the Period of Peak Lactation

Dietary NDF plays a crucial role as an essential nutrient for ruminants, serving as the most reliable indicator for cellulose expression. Ranathunga et al. [20] demonstrated a negative correlation between NDF and DMI when the primary source of NDF in the diet derives from forage. The current study expands upon this finding, observing a linear decrease in DMI alongside an increase in dietary NDF levels. Consequently, total NDF intake demonstrated a linear increase. These findings are consistent with the research conducted by Schulze [21], who also observed decreased DMI in dairy cows exposed to higher dietary NDF levels. Notably, the experimental group with 34% NDF exhibited significantly lower DMI compared to the 25% and 28% NDF groups, thereby negatively impacting the overall performance of the dairy cows.

Mertens [22] conducted a study to investigate the relationship between the physically effective peNDF of the diet, the pH value of the rumen fluid, and the milk fat rate. The study revealed that a peNDF level of 19.7% was necessary to maintain a milk fat rate of approximately 3.4%. The average pH value of the rumen fluid was 6.0, corresponding to a peNDF level of approximately 22.3%. Similarly, a meta-analysis by Zebeli et al. [23] suggested that the peNDF_8.0_ level in the diet should range from 14.9% to 18.5%, considering its effects on DMI, with 18.5% being a breakpoint value. In the current study, daily peNDF_8.0_ intake ranged from 2.68 kg to 4.01 kg, accounting for 11.22% to 17.69% of the DM intake. The peNDF_8.0_ levels of the 25% and 28% NDF groups were lower than Zebeli’s suggested level, because the two groups utilized non-forage fiber sources like beet pulp and whole cottonseed, effectively maintaining DMI and milk production.

Voelker et al. [24] compared different NDF levels in diets and found that daily eating time did not significantly differ between the 24% NDF and 31% NDF groups. However, daily ruminating time, chewing time, and average time spent eating, ruminating, and chewing per kilogram of DM were significantly higher in the 31% NDF group. Additionally, the average time spent eating and ruminating per kilogram of NDF was significantly higher in the 24% NDF group. Yang et al. [25] adjusted the particle size of the diet to achieve different peNDF contents and observed that increasing peNDF levels resulted in higher peNDF intake, daily rumination time, and chewing time, while daily eating time remained relatively stable. Beauchemin et al. [26] suggested that daily rumination time should account for more than 60% of the total chewing time to maintain rumen health. In the present study, as the dietary NDF levels increased from 25.49% to 34.69% daily eating and ruminating time in dairy cows gradually increased, these results were consistent with Voelker. Specifically, daily eating time increased by 43.99%, and daily ruminating time increased by 32.32%. The observed differences in daily eating and ruminating time in dairy cows, with a significant increase of 43.99% in daily eating time and 32.32% in daily ruminating time, were found to be statistically significant (*p* < 0.05).

In a study conducted by Mertens et al. [22] using 213 lactating cows, it was found that maintaining a milk fat rate of 3.6% required a daily chewing time of 744 min and 36.1 min spent chewing per kilogram of DM. For a milk fat rate of 3.4%, the corresponding values were 589 min and 27.7 min, while for 3.2% milk fat it took 479 min and 22.2 min, respectively. Sudweeks et al. [27] reported that maintaining a normal milk fat rate typically involves a chewing time of 30 min per unit of DM ingested, although some scholars suggest it to be 24 min. In the present study, an increase in dietary NDF levels resulted in a linear increase in the daily eating and ruminating time of dairy cows (*p* < 0.05). Specifically, as the NDF levels increased from 25.49% to 34.65% daily eating time increased by 43.99% and daily ruminating time increased by 32.32%, which aligns with previous research findings. Moreover, the average time spent eating and ruminating per kilogram of DM showed a linear increase (*p* < 0.05), indicating a close relationship with a significant decrease in DMI. Interestingly, the average time spent ruminating per kilogram of NDF and peNDF_8.0_ initially decreased and then increased, displaying a quadratic curve (*p* < 0.05). This could be attributed to the further increase in dietary NDF levels, particularly the breakthrough at 31% NDF content, resulting in a significant reduction in DMI. Consequently, cows compensate for the reduced nutrient intake by increasing the unit of nutrients consumed.

### 4.2. Effects of Dietary NDF Levels on Rumen Fermentation Parameters of Dairy Cows during Peak Lactation Period

#### 4.2.1. Rumen Average pH-Value, NH_3_-N and MCP

The concentration of dietary NDF has a significant positive correlation with the pH of rumen fluid. Insufficient NDF content in the diet leads to a significant reduction in rumination time, chewing time, and saliva secretion, resulting in a decrease in pH value [28]. In the present study, the 25% NDF test group showed significantly lower pH values compared to the 34% NDF test group (*p* < 0.05). This difference can be attributed to the higher NFC content in the 25% NDF group, leading to increased organic acid production. The average pH value of rumen fluid over 12 h showed a gradual increase with the increase in dietary NDF levels, with the 34% NDF test group displaying significantly higher values than the other three groups (*p* < 0.05). This finding aligns with previous studies by Jiang et al. [29] and Beauchemin [30].

Ruminal fluid NH_3_-N concentration reflects the balance between protein degradation and microbial protein synthesis from feed [31]. A low NH_3_-N concentration in the rumen fluid indicates slower ammonia release, which hampers bacterial protein synthesis by rumen microorganisms [32]. With the increase in dietary NDF levels, the concentration of NH_3_-N in the rumen fluid of dairy cows gradually decreased, and the 25% and 28% NDF test groups showed significantly higher concentrations compared to the 31% and 34% NDF test groups (*p* < 0.05). This can be attributed to the higher magnitude and rate of ammonia degradation in the 25% and 28% NDF groups compared to the 31% and 34% NDF groups. The decrease in NH_3_-N concentration is primarily due to a significant reduction in feed intake by dairy cows with higher NDF diets [6,8], resulting in decreased degradable protein in the feed. Additionally, increased NDF levels reduce the residence time of the diet in the rumen and accelerate feed flow [33], leading to a decreased protein degradation rate and subsequent decrease in NH_3_-N concentration.

The MCP in rumen fluid is primarily synthesized by rumen bacteria and protozoa using NH_3_-N. It is closely related to the rumen fermentation status and protein metabolism [34]. MCP provides 50% of small intestine absorbable protein for animals, and its output is influenced by adequate adenosine triphosphate (ATP) and nitrogen obtained from the degradation of non-protein and protein nitrogen sources [35]. In the current study, increasing the dietary NDF level significantly decreased the concentration of MCP in the rumen fluid of dairy cows (*p* < 0.05). This decrease can be attributed to the gradual reduction in NFC in the diet as NDF levels increase, leading to a decrease in readily available energy for rumen microorganisms and subsequently reducing energy–nitrogen synchronization, resulting in decreased MCP production.

#### 4.2.2. VFA and Lactate

The rumen of ruminant animals plays a crucial role in the production of VFA, primarily through the fermentation of dietary carbohydrates. These VFAs, namely acetate, propionate, and butyrate, are the major products of rumen fermentation, collectively contributing to approximately 95% of VFA production. Significantly, acetate and propionate serve as important precursors for the synthesis of milk fat and lactose, respectively [36]. Therefore, the ratio of acetate to propionate holds a close relationship with milk fat synthesis.

The composition of the diet significantly influences the type of rumen fermentation and the production of VFA. Research conducted by Demeyer et al. [37] demonstrated that an increase in crude fiber content within the diet promotes the proliferation of cellulolytic bacteria, resulting in enhanced acetate production and a shift towards acetate fermentation in the rumen. Similarly, Nocek et al. [38] found that an increase in starch content stimulates the growth of starch-decomposing bacteria, leading to increased propionate and lactate production and a subsequent shift towards propionate fermentation in the rumen. Tjardes et al. [39] and Liu et al. [40] conducted studies that showed a decrease in the concentration of total VFA, propionate, and butyrate in rumen fluid with increased levels of dietary NDF, while the concentration of acetate and the acetate to propionate ratio increased accordingly, which were consistent with these studies.

Lactate is a significant acid produced through rumen fermentation in ruminants. The concentration of lactate in ruminal fluid during episodes of subacute rumen acidosis in dairy cows has been the subject of varying reports. Keunen et al. [41] conducted research indicating that the concentration of lactic acid in the rumen does not exceed 10 mmo/L when subacute rumen acidosis occurs in dairy cows. Conversely, Beauchemin et al. [42] suggested that ruminal acidosis in dairy cows does not result in the presence of lactate in the rumen. In the present study, subacute rumen acidosis did not occur when the lactate concentration in the rumen of dairy cows ranged from 0.31 to 0.45 mmo/L, which further substantiates the low lactate content in the rumen.

#### 4.2.3. Rumen Cellulolytic Bacteria

Fibrobacter succinogenes, Ruminococcu albus, and Ruminococcu flavefaciens are recognized as the most prominent bacteria involved in fiber degradation within the rumen [43]. Studies conducted by Weimer et al. [44], Dehority et al. [45], Ghasemi et al. [46], and Saro et al. [47] have consistently demonstrated that an increase in dietary NDF levels corresponds to a gradual rise in the proportion of these three cellulolytic bacteria among the total bacterial population. This experimental finding aligns with the aforementioned research results. The abundance of these cellulolytic bacteria in this experiment followed the order: *Fibrobacter succinogenes* > *Ruminococcu albus* > *Ruminococcu flavefaciens*, which is consistent with the findings reported by Saro et al. [47]. However, the research conducted by Weimer et al. [44] and Cherdthong et al. [43] indicates that Ruminococcu albus constitutes the primary cellulolytic strain among the three, a disparity that might be attributed to the type of roughage in the diet and the ratio of concentrate to forage feed.

### 4.3. Effects of Dietary NDF Levels on the Production Performance of Dairy Cows during the Peak Lactation Period

The dietary composition and nutritional quality have a crucial impact on enhancing milk production and milk fat percentage in dairy cows. The energy intake serves as a direct determinant of dairy cow milk production and is influenced by the net lactation energy level and DMI of the diet. Carbohydrates, when acted upon by rumen microorganisms, yield a substantial quantity of VFAs, thereby meeting 70% to 80% of the energy requirements of dairy cows [48]. Among these fatty acids, acetate and propionate serve as crucial precursors for milk fat and lactose synthesis. The ratio of acetate to propionate plays a direct role in influencing the milk fat level.

Kanjanapruthipong et al. [49] conducted a study where dairy cows were fed diets with varying levels of NDF during the peak lactation period. The NDF levels tested were 24.90%, 27.77%, 30.56%, and 33.81%. The results showed that as the dietary NDF levels increased, there were significant reductions (*p* < 0.05) in milk production, 4% standard milk yield, milk protein rate and yield, and lactose yield. However, there was a significant increase (*p* < 0.05) in milk fat rate. Similarly, Jiang et al. [29] fed mid-lactation dairy cows diets with NDF levels of 35.4%, 38.0%, 41.9%, and 43.4% and observed similar trends. As the dietary NDF levels increased, there was a linear decrease in milk production, 4% fat-corrected milk (FCM), milk protein percentage, and yield. Conversely, there was a linear increase in milk fat percentage and feed efficiency (*p* < 0.05).

These findings suggest that higher NDF levels in the diet during the peak lactation period can lead to decreased milk production parameters such as milk yield, lactose yield, milk protein percentage, milk protein yield, and nitrogen efficiency while resulting in increased milk fat percentage and MUN concentration. However, the specific parameters of 4% fat-corrected yield, feed efficiency, and milk lactose percentage did not exhibit significant effects.

In this experiment, the increase in dietary NDF levels resulted in a gradual decrease in milk yield, milk protein percentage, milk protein yield, milk lactose yield, and nitrogen efficiency in dairy cows. At the same time, there was a gradual increase in milk fat percentage and MUN concentration, which significantly impacted milk yield, milk fat percentage, milk protein percentage, milk protein yield, milk lactose yield, MUN concentration, and nitrogen efficiency (*p* < 0.05). The reductions in milk production were consistent with the corresponding reductions in DMI.

The observed effects of dietary NDF on milk fat percentage align with the findings of the aforementioned studies. Higher NDF content in the diet promotes the synthesis of acetate, which is a precursor for milk fat synthesis. Milk lactose yield showed a linear decrease (*p* < 0.05) associated with the decrease in ruminal propionate concentration as dietary NDF levels increased. Propionate serves as a precursor for glucose synthesis. Nitrogen efficiency, milk protein percentage, and milk protein yield also demonstrated a gradual decrease with increased dietary NDF levels. The decrease in nitrogen efficiency and milk protein yield (*p* < 0.05) can be attributed to the diminishing proportion of NFC in the diet, which reduces the availability of rapidly fermentable energy in the rumen. This, in turn, reduces the synchronization of energy and nitrogen, leading to a decline in nitrogen utilization efficiency and milk protein rate. The concentration of milk urea nitrogen significantly increased with higher dietary NDF levels (*p* < 0.05); this difference may be explained by the decreased energy intake. Adequate energy improves rumen microbial protein production and NH_3_ utilization, which decreases rumen NH_3_, BUN (blood urea nitrogen), and MUN [50].

In conclusion, the above findings demonstrated that increasing dietary levels of NDF during the peak lactation period have significant effects on milk production and composition in dairy cows. Higher NDF levels resulted in reduced milk yield, protein percentage, and lactose yield while increasing milk fat percentage. Nitrogen efficiency and milk protein production decline with higher NDF levels, likely due to decreased availability of rapidly fermentable energy.

## 5. Conclusions

Based on the experiment’s findings, it is clear that the NDF level in the diet significantly influences various aspects of dairy cow performance during peak lactation. Setting the NDF level at 25% resulted in adverse effects on daily feeding and rumination time, rumen pH value, acetate concentration, milk fat percentage, and milk fat production. These results indicate that a lower NDF level negatively affects rumen health and milk quality. Conversely, increasing the NDF level to 34% led to significant reductions in DMI, rumen TVFA concentration, MCP yield, milk yield, milk protein rate, and milk protein yield, as well as nitrogen efficiency. Additionally, there was a notable increase in MUN concentration. Comparing the diets with NDF levels of 28% and 31%, the 28% NDF diet showed more favorable results in terms of feed intake, rumen fermentation indices (MCP, TVFA, and Propionate), and overall production performance (milk yield, milk protein yield, MUN, and nitrogen efficiency). Consequently, it is recommended to feed dairy cows with a diet containing 28% NDF during the peak lactation period to optimize milk production outcomes.

## Figures and Tables

**Table 1:** Nutrient levels of main NDF ingredients ^1^ (% DM).

Ingredients	DM	CP	NDF	ADF	EE	Ca	P	Ash
Corn silage	26.65	8.80	47.39	28.24	2.40	0.31	0.27	22.33
Imported alfalfa	93.69	19.20	38.63	29.58	2.50	1.47	0.28	10.85
Local alfalfa	93.33	14.50	52.21	40.63	1.10	1.30	0.04	7.68
Leymus chinensis	92.66	7.16	70.00	40.03	3.90	0.40	0.20	5.62
Local oat hay	93.08	9.10	64.38	38.60	1.60	0.43	0.36	16.42

^1^ DM is dry matter; CP is crude protein; NDF is neutral detergent fiber; ADF is acid detergent fiber; EE is ether extract, Ca is calcium; P is phosphorus.

**Table 2:** Composition and nutrient levels of the experiment diets ^1^ (dry matter basis).

Items	Treatments			
	25.49%	28.65%	31.66%	34.65%
Ingredients, %				
Corn silage	18.00	18.00	16.00	10.00
Imported alfalfa hay	15.00	10.00	6.00	2.00
Local alfalfa hay	5.00	6.00	8.00	6.00
Leymus chinensis	1.00	4.00	8.00	12.00
Local oat hay	1.00	2.00	4.00	10.00
Corn grain	17.99	15.99	13.99	13.52
Steam-flaked Corn	13.26	11.47	9.00	7.00
Extruded soybean	3.18	3.18	3.18	3.18
Soybean meal	10.20	10.20	10.70	11.30
Rapeseed meal	5.08	5.08	5.08	5.08
Apple pomace	1.60	1.60	1.60	2.60
Beet pulp	1.68	1.68	1.68	1.68
Whole Cottonseed	0.88	4.46	5.79	8.33
Bergafat T 300	1.32	1.42	2.02	2.22
Molasses	1.12	1.12	1.12	1.12
Premix	3.69	3.80	3.84	3.97
Total	100.00	100.00	100.00	100.00
DM, %	52.40	52.32	52.20	52.14
NE_L_, Mcal/kg	1.68	1.68	1.67	1.69
CP, %	17.02	16.96	16.95	16.97
NDF, %	25.49	28.65	31.66	34.65
ADF, %	16.88	18.81	20.54	22.14
NFC, %	45.02	41.54	38.03	34.84
Starch, %	27.92	25.42	21.99	19.23
EE, %	5.17	5.85	6.65	7.22
Ca, %	0.87	0.87	0.87	0.87
P, %	0.34	0.34	0.34	0.34

^1^ DM is dry matter; NE_L_ is net energy of milk production; CP is crude protein; NDF is neutral detergent fiber; ADF is acid detergent fiber; NFC is non-fiber carbohydrate; EE is ether extract, Ca is calcium; P is phosphorus; the premix contains: vitamin A 92,000 IU, vitamin D_3_ 23,100 IU, vitamin E 840 IU, nicotinic acid 84 mg, copper 330 mg, manganese 504 mg, zinc 1306 mg, iodine 13 mg, selenium 16 mg, and cobalt 10 mg; NE_L_ value was calculated, while the others were measured; NFC = 100 − (CP + NDF + EE + ASH).

**Table 3:** The primers of real-time PCR.

Gene Name	Forward/Reward Primer	Base Number	Product Size
General bacteria	F:CGGCAACGAGCGCAACCC	18	146 bp
	R:CCATTGTAGCACGTGTGTAGCC	22	
Ruminococcu flavefaciens	F:CGAACGGAGATAATTTGAGTTTACTTAGG	29	132 bp
	R:CGGTCTCTGTATGTTATGAGGTATTACC	28	
Ruminococcu albus	F:CCCTAAAAGCAGTCTTAGTTCG	22	176 bp
	R:CCTCCTTGCGGTTAGAACA	19	
Fibrobacter succinogenes	F:GTTCGGAATTACTGGGCGTAAA	22	121 bp
	R: CGCCTGCCCCTGAACTATC	19	

**Table 4:** Effects of dietary NDF levels on DM, NDF, and peNDF intake in dairy cows during peak lactation ^1^.

Items	Treatment				SEM		*p* Value	
	25%	28%	31%	34%		All	L	Q
Feed intake, kg/d								
DM	23.89 ^a^	23.66 ^a^	23.28 ^ab^	22.67 ^b^	0.19	0.02	<0.01	0.37
NDF	6.09 ^d^	6.78 ^c^	7.37 ^b^	7.85 ^a^	0.08	<0.01	<0.01	0.23
peNDF_8.0_	2.68 ^d^	3.25 ^c^	3.62 ^b^	4.01 ^a^	0.03	<0.01	<0.01	0.05

^a, b, c, d^ Mean with different superscripts differ significantly (Duncan’s test, *p* < 0.05); ^1^ SEM is the standard error of the mean; DM is dry matter; NDF is neutral detergent fiber; peNDF is physically effective NDF.

**Table 5:** Effects of dietary NDF levels on eating and ruminating activities of dairy cows during peak lactation ^1^.

Feeding Behavior	Treatment				SEM	*p* Value		
	25%	28%	31%	34%		All	L	Q
Eating Time								
min/d	181.58 ^c^	215.78 ^b^	253.53 ^a^	261.46 ^a^	4.58	<0.01	<0.01	0.03
min/kg of DM	7.60 ^c^	9.13 ^b^	10.89 ^a^	11.53 ^a^	0.20	<0.01	<0.01	0.07
min/kg of NDF	29.82 ^c^	31.89 ^bc^	34.41 ^a^	33.28 ^ab^	0.67	0.01	0.01	0.05
min/kg of peNDF_8.0_	67.70 ^ab^	66.44 ^ab^	70.22 ^a^	65.26 ^b^	1.36	0.16	0.56	0.23
Ruminating time								
min/d	371.06 ^b^	379.25 ^b^	411.75 ^b^	491.00 ^a^	12.35	<0.01	<0.01	0.03
min/kg of DM	15.56 ^c^	16.04 ^bc^	17.70 ^b^	21.66 ^a^	0.52	<0.01	<0.01	0.02
min/kg of NDF	61.05	56.00	55.94	62.53	2.03	0.13	0.65	0.03
min/kg of peNDF_8.0_	138.75 ^a^	116.67 ^b^	114.16 ^b^	122.62 ^b^	4.45	0.03	0.04	0.01

^a, b, c^ Mean with different superscripts differ significantly (Duncan’s test, *p* < 0.05). ^1^ SEM is the standard error of the mean; DM is dry matter; NDF is neutral detergent fiber; peNDF is physically effective NDF.

**Table 6:** Impact of dietary NDF levels on rumen pH, NH_3_-N, MCP, lactate, and VFA in dairy cows during peak lactation ^1^.

Item	Treatment				SEM	*p* Value		
	25%	28%	31%	34%		All	L	Q
Mean pH	6.13 ^b^	6.25 ^b^	6.29 ^b^	6.51 ^a^	0.05	0.01	<0.01	0.36
NH_3_-N, mg/dL	11.50 ^a^	10.88 ^b^	9.83 ^b^	9.48 b	0.29	<0.01	<0.01	0.60
MCP, mg/dL	104.52 ^a^	103.10 ^a^	100.03 ^b^	91.74 ^c^	0.67	<0.01	<0.01	<0.01
Lactate, mmol/L	0.39 ^a^	0.38 ^a^	0.34 ^b^	0.32 ^b^	0.01	<0.01	<0.01	0.27
VFA, mmol/L								
Total VFA	103.89 ^a^	102.27 ^ab^	99.56 ^ab^	98.75 ^b^	1.25	0.09	0.02	0.76
Acetate (A)	59.12 ^b^	59.21 ^b^	60.82 ^a^	61.52 ^a^	0.38	0.01	<0.01	0.48
Propionate (P)	28.28 ^a^	27.71 ^a^	24.39 ^b^	23.94 ^b^	0.54	<0.01	<0.01	0.91
Butyrate	11.16 ^a^	10.31 ^ab^	9.52 ^ab^	8.75 ^b^	0.61	0.12	0.03	0.95
Isobutyrate	1.00	1.04	0.98	0.96	0.03	0.45	0.29	0.42
Isovalerate	2.91	2.68	2.55	2.38	0.15	0.19	0.04	0.84
Caproate	1.43	1.33	1.31	1.21	0.06	0.23	0.06	0.99
A:P	2.10 ^b^	2.14 ^b^	2.50 ^a^	2.57 ^a^	0.05	<0.01	<0.01	0.81

^a, b^ Mean with different superscripts differ significantly (Duncan’s test, *p* < 0.05); ^1^ SEM is the standard error of the mean; NH_3_-N is ammonia nitrogen; MCP is microbial crude protein; VFA is volatile fatty acid.

**Table 7:** Impact of dietary NDF levels on the proportion of rumen cellulolytic bacteria among total bacteria in dairy cows during peak lactation period (%).

Item	Treatments				SEM	*p* Value		
	25%	28%	31%	34%		All	L	Q
Fibrobacter succinogenes	0.1425 ^b^	0.1966 ^ab^	0.2113 ^a^	0.2240 ^a^	0.01	0.02	<0.01	0.16
Ruminococcu albus	0.1291	0.1303	0.1366	0.1375	0.02	0.98	0.75	0.99
Ruminococcu flavefaciens	0.0112 ^b^	0.0139 ^ab^	0.0149 ^ab^	0.0187 ^a^	<0.01	0.16	0.04	0.79

^a, b^ Mean with different superscripts differ significantly (Duncan’s test, *p* < 0.05).

**Table 8:** Effects of dietary NDF levels on dairy cows Milk yield and compositions of dairy during the period of peak lactation ^1^.

Item	Treatments				SEM	*p* Value		
	25%	28%	31%	34%		All	L	Q
Milk yield, kg/d	43.08 ^a^	42.16 ^ab^	40.42 ^ab^	37.68 ^b^	1.28	0.09	0.02	0.5
4% FCM yield, kg/d	41.81	40.58	39.69	39.65	1.38	0.67	0.28	0.68
Feed efficiency	1.75	1.71	1.71	1.75	0.06	0.93	0.99	0.53
Milk fat percentage, %	3.83 ^b^	3.99 ^ab^	4.15 ^ab^	4.36 ^a^	0.11	0.03	0.01	0.05
Milk lactose percentage, %	5.01	4.95	4.90	4.95	0.06	0.67	0.42	0.41
Milk protein percentage, %	3.14	3.06	3.01	2.94	0.06	0.18	0.04	0.86
Milk fat yield, kg/d	1.64	1.69	1.68	1.64	0.06	0.03	0.01	0.05
Milk lactose yield, kg/d	2.15 ^a^	2.09 ^a^	1.98 ^ab^	1.86 ^b^	0.06	0.04	0.01	0.67
Milk protein yield, kg/d	1.34 ^a^	1.29 ^a^	1.21 ^ab^	1.11 ^b^	0.04	0.02	0.01	0.58
MUN, mg/dL	11.23 ^c^	12.72 ^a^	14.25 ^b^	14.51 ^a^	0.37	<0.01	<0.01	0.15
Nitrogen efficiency, %	0.33 ^a^	0.32 ^ab^	0.30 ^ab^	0.29 ^b^	0.01	0.13	0.03	0.92

^a, b, c^ Mean with different superscript differ significantly (Duncan’s test, *p* < 0.05); ^1^ SEM is the standard error of the mean; 4.0% FCM yield = (0.4 × kg of milk) + (15.0 × kg of fat); Feed efficiency = 4% FCM yield/DMI; ^3^ MUN is milk urea nitrogen; Nitrogen efficiency = (milk protein yield (kg/d) ÷ 6.38) / (crude protein intake (kg/d) ÷ 6.25).

## Data Availability

All the data used in the current article are available in the submitted version of the article.
